# De Novo Transcriptome Analysis of Differential Functional Gene Expression in Largemouth Bass (*Micropterus salmoides*) after Challenge with *Nocardia seriolae*

**DOI:** 10.3390/ijms17081315

**Published:** 2016-08-11

**Authors:** Omkar Byadgi, Chi-Wen Chen, Pei-Chyi Wang, Ming-An Tsai, Shih-Chu Chen

**Affiliations:** Department of Veterinary Medicine, College of Veterinary Medicine, National Pingtung University of Science and Technology, Pingtung 91201, Taiwan; omkarcof1@gmail.com (O.B.); e6261816@yahoo.com.tw (C.-W.C.); p9416004@mail.npust.edu.tw (M.-A.T.)

**Keywords:** Illumina paired-end sequencing, immune response, largemouth bass (*Micropterus salmoides*), *Nocardia seriolae*, transcriptome

## Abstract

Largemouth bass (*Micropterus salmoides*) are common hosts of an epizootic bacterial infection by *Nocardia seriolae*. We conducted transcriptome profiling of *M. salmoides* to understand the host immune response to *N. seriolae* infection, using the Illumina sequencing platform. De novo assembly of paired-end reads yielded 47,881 unigenes, the total length, average length, N50, and GC content of which were 49,734,288, 1038, 1983 bp, and 45.94%, respectively. Annotation was performed by comparison against non-redundant protein sequence (NR), non-redundant nucleotide (NT), Swiss-Prot, Clusters of Orthologous Groups (COG), Kyoto Encyclopaedia of Genes and Genomes (KEGG), Gene Ontology (GO), and Interpro databases, yielding 28,964 (NR: 60.49%), 36,686 (NT: 76.62%), 24,830 (Swissprot: 51.86%), 8913 (COG: 18.61%), 20,329 (KEGG: 42.46%), 835 (GO: 1.74%), and 22,194 (Interpro: 46.35%) unigenes. Additionally, 8913 unigenes were classified into 25 Clusters of Orthologous Groups (KOGs) categories, and 20,329 unigenes were assigned to 244 specific signalling pathways. RNA-Seq by Expectation Maximization (RSEM) and PossionDis were used to determine significantly differentially expressed genes (False Discovery Rate (FDR) < 0.05) and we found that 1384 were upregulated genes and 1542 were downregulated genes, and further confirmed their regulations using reverse transcription quantitative PCR (RT-qPCR). Altogether, these results provide information on immune mechanisms induced during bacterial infection in largemouth bass, which may facilitate the prevention of nocardiosis.

## 1. Introduction

During intensive aquaculture, fish are always exposed to stressors which may facilitate host infection by opportunistic pathogens existing in the water [1]. Indeed, detecting the invading pathogens depends on the host’s ability to recognize the pathogens [2,3]. Therefore, for rapid elimination of pathogens, fish rely on innate or nonspecific immune responses [1]. Against this background, transcriptome profiling analysis during infection in the host can facilitate genome studies and functional gene identification. However, in fish the broad identification of immune-related genes at the genome or transcriptome levels are limited to a few species [4,5]. Since the genome sequence for many non-model fish species is unknown, the study on immune genes is difficult. Moreover, the introduction of RNA deep sequencing technologies (i.e., Solexa/Illumina RNA-Seq and digital gene expression) have contributed much to the identification of important immune-related genes in fish [6,7,8,9].

In this study, we concentrated on *Nocardia seriolae*, a Gram-positive, acid-fast bacterium with branched hyphae which causes nocardiosis in cultured marine and freshwater fish in Taiwan, Japan, and China [10,11,12,13,14,15,16]. *Nocardia seriolae* infections frequently result in considerable economic loss to fish farmers in Taiwan. Recently, after infection with pathogenic microorganisms *Aeromonas hydrophila* in zebrafish (*Danio rerio*) [17] and *Vibrio anguillarum* infection in sole (*Cynoglossus semilaevis*) [18], the transcriptome profile has been reported. Additional examples of transcriptome profiling analyses include the orange-spotted grouper (*Epinephelus coioides*) [19], blunt snout bream (*Megalobrama amblycephala*) [20], Chilean abalone *Concholepas* (*Gastropoda*, *Muricidae*) [21], grass carp (*Ctenopharyngodon idella*) [22], blowfish or fugu (*Takifugu rubripes*) [23], large yellow croaker (*Larimichthys crocea*) [24], and Nile tilapia (*Oreochromis niloticus*) [25,26]. Several studies have also reported transcriptome profiles for *L.* *crocea* in response to immune stimuli, pathogenic infection, or environmental stress [27,28,29]. However, to our knowledge there are no studies related to fish transcriptomes for identification of gene expression profiles in response to *Nocardia seriolae* infection. In this study, we assembled the transcriptome of largemouth bass (*M. salmoides*) spleen and compared the gene expression profiles among *Nocardia seriolae*-infected and control groups to exhibit the molecular fitness mechanisms against bacterial infection and frame a possible strategy to prevent the outbreak of nocardiosis.

## 2. Results

### 2.1. Transcriptome Sequence Assembly

Of 47,881 unigenes, 37,712 (78.76%) were annotated using at least one database, including 36,686 (97.27%) in NT, 28,964 (76.80%) in NR, 24,830 (65.81%) in Swiss-Prot, 8913 (23.63%) in KOG, 20,329 (53.90%) in KEGG, 22,194 in Interpro (58.85%), and 835 (2.21%) in GO (Tables S1 and S2).

### 2.2. Functional Classification

**Figure 1:**
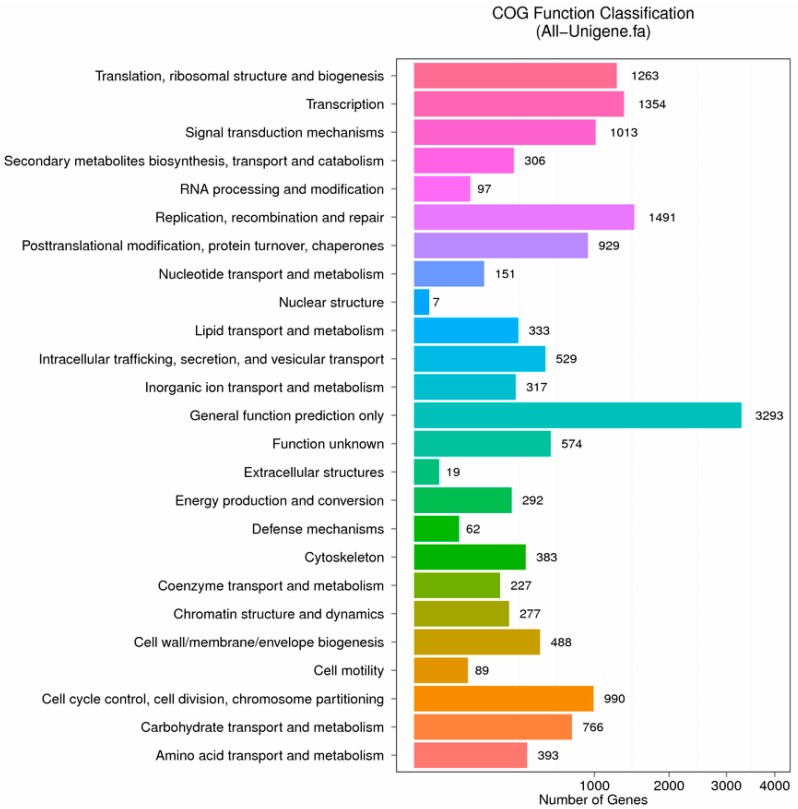
The cluster of orthologous groups (COG) classification. 8913 (23.63% of the total annotated putative proteins) were grouped into 25 different categories.

Overall, 8913 (23.63%) annotated putative proteins from COG were grouped into 25 different categories (Figure 1). After filtering the poorly characterised proteins (“general function prediction only” and “function unknown”) based on the number of unigenes, the top three functional clusters were determined to be “replication recombination and modification” (1491, 16.72%), which is followed by “transcription” (1354, 15.19%) and “translation, ribosomal structure, and biogenesis” (1263, 14.17%) (Figure 1).

**Figure 2:**
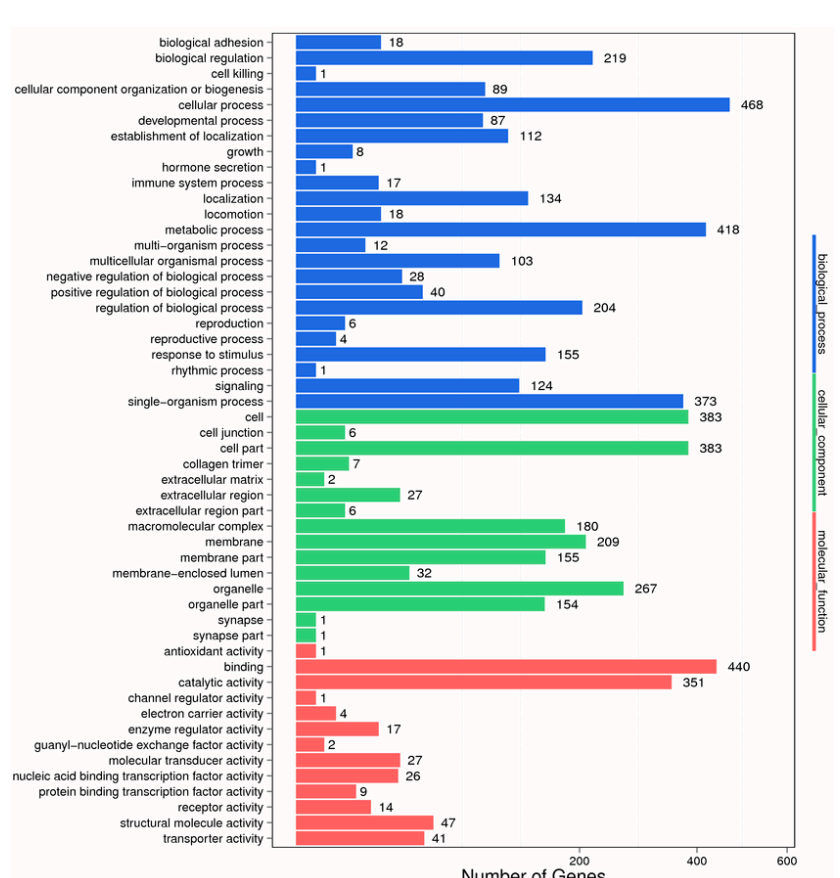
Functional distribution of GO annotation.

Furthermore, 37,712 (78.76%) unigenes were assigned to 835 GO terms based on sequence homology and a total of 52 functional groups were clustered into biological process, cellular component, and molecular function (Figure 2). The unigene sequences from molecular function were clustered into 13 different classifications. Further, the largest subcategory within molecular function was “binding”, followed by “catalytic activity” In the biological process, sequences were distributed into 24 classifications. The most represented subcategories were “cellular processes” and “metabolic processes”. “Cell part” and “cell” were the most represented among 13 subcategories within the cellular component category.

**Figure 3:**
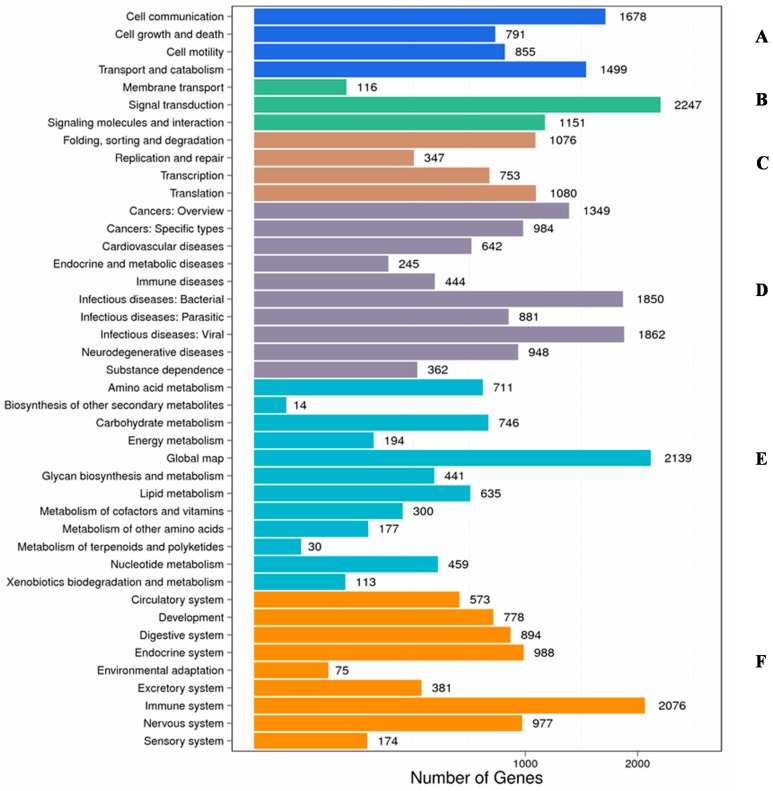
KEGG classification of assembled unigenes from control and treated groups. (**A**) Cellular processes; (**B**) Environmental information processing; (**C**) Genetic information processing; (**D**) Human diseases; (**E**) Metabolism; and (**F**) Organismal systems.

Overall, 20,329 (53.90%) sequences had significant matches were allocated to 244 KEGG pathways. Moreover, the highest number of genes categorised from KEGG analysis related to human disease accounted for 9567 (28.10%) genes, with sub-groups from bacterial infectious diseases (1850 genes), viral infectious diseases (1862 genes), and cancer-related genes (1349 genes). Further, 6919 (20.32%) genes were related to organismal systems where the majority of the genes were categorised as immune system-related (2076 genes), followed by endocrine system-related (988 genes), nervous system-related (977 genes), digestive system-related (894 genes), and development-related (778 genes) (Figure 3). Subsequently, metabolism, cellular processes, environmental information processing, and genetic information processing accounted for 5959 (17.50%), 4823 (14.17%), 3514 (10.32%), and 3256 (9.56%) genes, respectively.

### 2.3. Differentially Expressed Genes after Nocardia seriolae Challenge

**Figure 4:**
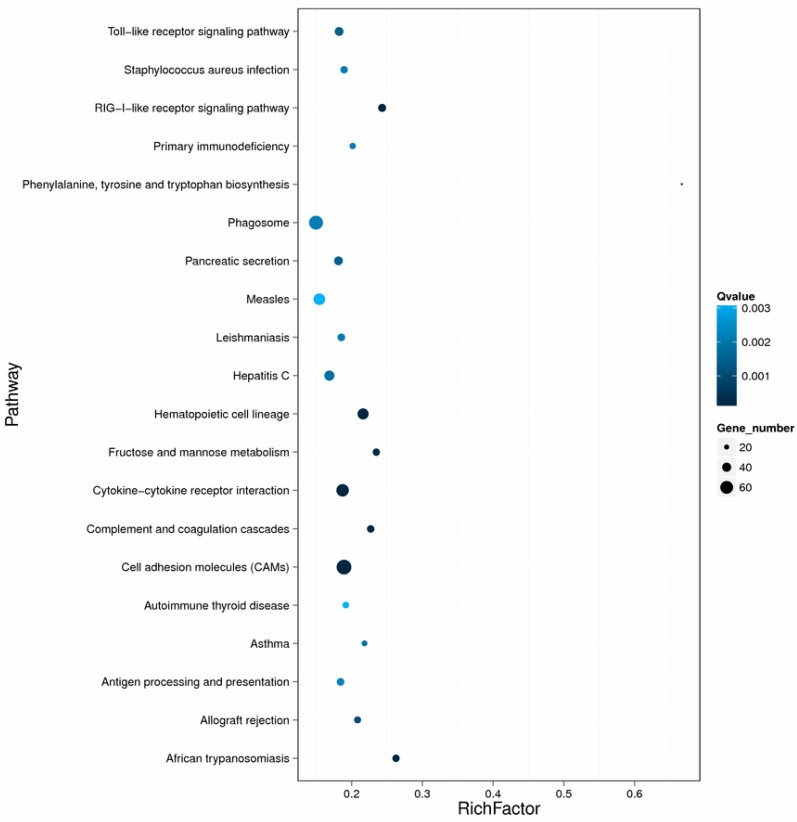
Scatterplot of the top 20 enriched KEGG pathways. Rich Factor is the ratio of differentially expressed gene numbers annotated in this pathway terms to all gene numbers annotated in this pathway term. *q* ≤ 0.05 as significantly enriched.

A total of 1384 transcript-derived unigenes were upregulated, whereas 1542 genes were downregulated in phosphate buffered saline (PBS) control and bacterial infection groups, respectively (Figure S1). The top 20 enriched pathways are shown in Figure 4, with genes involved in immune-related “Cell adhesion molecule”, “Cytokine receptor interaction”, “Hematopoietic cell lineage”, and “Phagosome” categories being the most significantly enriched. Natural killer cell-mediated cytotoxicity, hematopoietic cell lineage, toll-like receptor signalling, Fc γ R-mediated phagocytosis, antigen processing and presentation, NOD-like receptor signalling, and chemokine signalling (Table S3) were differentially expressed3among immune-related categories. These results suggest an important role for these unigenes during *N. seriolae* infection in largemouth bass.

The differential expression in immune-related genes were identified from 13 pathways (Table S3) and were mapped to the KEGG database and observed their association among cytokines and their receptors (e.g., IL6, IL8, IL8R, IL4R, IL13RA1, IL12RB2, CXCL12, CXCR4, CCR5), toll-like receptor signalling (TLR) pathways (Figure S2) (e.g., LBP, CASP8, IKK γ, IKK α, IKK β, TRAF6, RIP1, CTSK, TLR3, IFN-αβR, IKKε, STAT1, IRF3, IRF7, p38, TNF α, IL1β, IL12, IL8, RANTES, CD40, CD86, IP10), and T cell receptor signalling (e.g., TCR, CD3, CD4/8, CD28). *N. seriolae* infection also influenced genes significantly related to transcriptional regulation, including NF-κB signalling (Figure S3) (NEMO, TRIM25, IKBKG, and RIP1), and JAK-STAT signalling (Figure S4) (STAM, STAT1, SOCS, and SHP2). Unigenes representative of genes differentially expressed during bacterial infection are listed in Table 1.

### 2.4. Differentially Expressed Gene Validation Using Real-Time PCR

**Figure 5:**
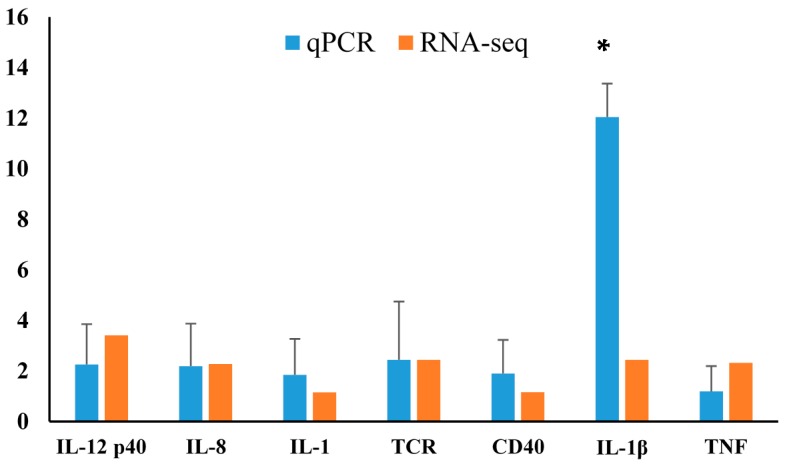
Comparative gene expression analysis from qPCR and RNA-Seq in spleen from the infected largemouth bass with *N. seriolae* and compared with those in the control at the 24 h time point. Expression of target genes was normalized to *β-actin* as a reference gene. Statistically significant differences from control are presented, with * *p* < 0.05.

We identified immune-related gene sequences that were upregulated from DEG in largemouth bass (Table S4), and evaluated their homology with those from other fish species using the NCBI database. These sequences will be used for our future studies in immune response of largemouth bass to *Nocardia seriolae*. The expression levels of seven differentially expressed genes related to pathways including TLR, RIG I-like receptors, cytokine-cytokine receptor interaction, natural killer cell mediated cytotoxicity, and antigen processing and presentation (T-cell receptor (TCR)) were evaluated from spleen tissue. The expression levels were largely consistent with the transcriptome profile analyses suggesting that the transcriptome data were reliable (Figure 5).

## 3. Discussion

In the present study, Illumina sequencing of control and infection treatment groups yielded 47,881 merged unigenes from spleen tissue of largemouth bass (*M. salmoides*). This study selected the spleen of largemouth bass 24 h after challenge as experimental samples. After challenge with *N. seriolae* we observed upregulations of many immune-related genes in the largemouth bass. Noticeably, immune-related pro-inflammatory cytokines and signal transduction related genes, including IL-1β, TNF receptor, CXC chemokine, TGF-β, and NF-κB, were the most significantly upregulated transcripts.

After assembly, 47,881 unigenes were generated with an average length of 1038 bp and an N50 of 1983 bp, longer than the sequences achieved in previous studies using a Roche GS FLX 454 system (Basel, Switzerland) with a MIRA assembler [30] or an Illumina/Hiseq-2000 with assembling program SOAP [31]. This difference in sequence quality may be explained by differences in the sampling tissue and de novo assemblers. Since largemouth bass has an absence of a reference genome in the database, the Trinity program used in this study showed better performance compared to other tools in transcriptome assembly [32,33]. In contrast to Trinity, SOAP or MIRA assemblies adopted in previous studies [30,31] have been shown to be more fragmented with high levels of errors in sequencing and polymorphism [33,34]. In this study, the largemouth bass transcriptome yielded 47,881 merged unigenes from the Illumina/Hiseq-2000 RNA-Seq platform compared to 29,682 unigenes from the Roche 454 system and 2139 unigenes from a SMART cDNA library [35].

It is noteworthy that only 37,712 unigenes were annotated from the databases in this study based on sequence similarity; this annotation limitation also exists in other marine organism transcriptomes [36]. This could be explained due to the absence of a genomic database and genomic studies on commercially important aquaculture species [32,37,38,39]. The GO, COG, and KEGG databases used in this study for functional annotation provide valuable information about biological features of largemouth bass challenged by *N. seriolae*. For example, in the KEGG analysis of 20,329 sequences assigned to 244 KEGG pathways, genetic information processing accounted for 9567 pathways related to pathogen infection (Figure 3). Together, these findings indicate that primary host immune pathways are conserved in largemouth bass which are activated to protect against pathogen infections.

Cytokines are proteins which transfer information among cells to initiate complex intracellular biological processes upon binding to corresponding cell-surface receptors. Moreover, cytokine levels initiate an inflammatory response to bacterial exposure which guides towards leukocyte attraction and activation of antimicrobial pathways [40,41]. Against this background, tumour necrosis factor alpha (TNF-α), which is a first cytokine released during infection activates the downstream expression of other cytokines such as IL-1β and chemokines [42,43]. In the present study, after *N. seriolae* infection it was observed that different cytokines and cytokine receptor families are upregulated in cytokine–cytokine receptor interaction signalling pathways (Table 1), including chemokine receptors (CXCL10, CXCR3, XCR1, CCL 20, 25, 19, 21, 5, CCR3), hematopoietin receptors (IL11RA IL6R), TNF receptors (SF11B, TNFSF12, SF14, and SF6B), TGF-β receptors (TGFBR2), and IL-1 receptors (IL-1β, IL-18, and IL-1R1). These data indicate that, in the case of largemouth bass in early stages of *N. seriolae* infection, cytokine–cytokine receptor interaction may represent an important anti-bacterial mechanism.

In the host, pattern-recognition receptors (PRRs) recognise pathogen-associated molecular patterns (PAMPs) to defend against pathogen invasion and activate immune responses through signalling pathways, such as TLRs, RIG-I-like receptors (RLRs), NOD-like receptors (NLRs) [44], and C-type lectin receptors (CLRs) [45,46]. In this study, a total of 29 gene transcripts, which are involved in the TLR signalling pathway, are found to be upregulated, including the fish-specific TLRs (TLR22), and downstream effector molecules, such as LBP, CASP8, IKK α, IKK β, TRAF6, TAK, TBK, IKK, and RANTES. Additionally, we observed downstream effector molecules of cytokines and transcription factors including p38, IRF3, IRF7, STAT1, IL-12, IL-8, CD40, CD86, and IP10. These suggest that TLR mechanisms are conserved from fish to mammals. We observed upregulations in the expression of pro-inflammatory cytokines in our study after *N. seriolae* infection including IL-1β, IL-8, and TNF-α (Figures S2 and S3). Our results on TNF-α and IL-1β were in agreement with the study on Japanese flounder (*Paralichthys olivaceus*) in spleen after immersion challenge with *N. seriolae*, wherein TNF-α and IL-β were upregulated at 24 h post challenge, while CC chemokine downregulated [47]. Moreover, in the case of human monocytes, cytokines induced within 24 h following Gram-positive and Gram-negative bacterial infections [48].

The Janus kinase/signal transducers and activators of transcription (JAK-STAT) pathway initiated due to interleukins, IFNs, and growth factors present in the surrounding microenvironment [49]. Different cytokine receptors are associated with JAK for proliferation, survival, and differentiation in lymphoid cell precursor [50,51], while STAT1 activated upon IFN-γ signalling, resulting in enhanced bacteria killing and protection [48]. In this study, the members of the JAK-STAT, including *STAM* and *Stat1*, were upregulated (Figure S4). This can suggest that, the JAK-STAT pathway activated upon *N. seriolae* infection in largemouth bass, which can further induce other pathways, namely NF-κB signalling, the TGF-β activated SMAD pathway, and apoptosis [52].

## 4. Materials and Methods

### 4.1. Animal Maintenance

Healthy largemouth bass (*Micropterus salmoides*) without pathogen infection weighing 125 ± 10 g were used in this study. The fish were kept in an indoor facility at a constant temperature of 26 °C and fed daily with commercial feed. The experiment was performed two weeks after acclimatisation. Fish were anaesthetised for handling with 2-phenoxyethanol. Approval for the following animal studies was obtained from the Centre for Research Animal Care and Use Committee of the National Pingtung University of Science and Technology under protocol number 101-027, dated 19 March 2012.

### 4.2. Isolation, Cultivation, and Challenge with Nocardia seriolae

The bacterium *N. seriolae* was isolated from striped bass and found to be highly virulent in farmed fish [53]. The species was identified by API ZYM and 16S rDNA sequencing, grown in Brain Heart Infusion (BHI) broth for five days at 25 °C, and enumerated prior to the challenge test. Fifteen fish were anaesthetised and injected intraperitoneally with 1.0 × 10^6^ cfu *N. seriolae* that were suspended in 100 μL phosphate-buffered saline (PBS, pH 7.2). The remaining 15 fish per group received only PBS (pH 7.2) as a control. After the fish were returned to the observation tanks, samples were taken at 24 h post infection (hpi). Three fish each from the challenge (treatment) and control groups (*n* = 3) were examined. Spleen tissue was dissected and total RNA was isolated.

### 4.3. Total RNA Extraction, Preparation of cDNA Library, and Sequencing

Total RNA was extracted using TRIzol^®^ reagent (Invitrogen Corp., Carlsbad, CA, USA). RNA integrity was assessed using Agilent Bioanalyzer 2100 system (Agilent Technologies, Palo Alto, CA, USA). A TruSeq™ RNA Sample Preparation Kit (Illumina, Inc., San Diego, CA, USA) was used for cDNA library construction. Further, 40 μg total RNA was used for mRNA isolation using poly-T oligo-attached magnetic beads. First-strand cDNA was synthesized using random hexamer primers and Superscript III (Invitrogen, Carlsbad, CA, USA); this was followed by second-strand cDNA synthesis, end repair, and adaptor ligation. The RNA-Seq library was sequenced on the Illumina HiSeq™ 2000 (Illumina, Inc., San Diego, CA, USA) platform as paired-end reads to 100 bp at Genomics Bioscience Technology Co., Ltd. (Taipei, Taiwan). The transcriptome raw sequencing datasets are available from Sequence Read Archive (SRA) database in NCBI and the accession numbers are SRX1739692 and SRX1738842. All of the information on the assembled unigene sequences and annotations are available from the corresponding authors upon request.

### 4.4. Filtering of Sequencing Reads

Raw reads were defined as adaptor-polluted reads containing low-quality or unknown base (N) reads; these reads were removed before downstream analyses. Internal software was used to filter reads, removing (1) reads with adaptors; (2) reads in which unknown bases comprised greater than 5% of the read; and (3) low quality reads (defined as the percentage of bases for which quality is less than 10 and greater than 20% in a read). After filtering, the remaining reads were called “Clean Reads” and stored in FASTQ [54] format.

### 4.5. De Novo Transcriptome Assembly

Trinity [55] was used to perform de novo assembly with clean reads. Next, TIGR Gene Indices clustering tools, or Tgicl, was used to cluster transcripts to unigenes. In the case of two or more samples, Tgicl would be re-executed with each sample’s unigene to obtain the final unigene for downstream analysis. Unigenes were divided into two classes: clusters (CL), comprised of several unigenes with shared similarity greater than 70%, and singletons (Unigenes).

### 4.6. Functional Unigene Annotation and Classification

For gene annotation, following database were used; NCBI non-redundant protein database [56], gene ontology (GO) [57], Clusters of Orthologous Groups [58], and the Kyoto Encyclopaedia of Genes and Genomes [59] with E-values less than 10^−5^ using BlastP (Version 2.2.25) [60]. With functional annotation, we selected the region of the unigene that best mapped to functional databases in a priority order of NR, SwissProt, KEGG, and COG as its coding sequence (CDS), and displayed this sequence region from 5’ to 3’ in FASTA format. Unigenes that could not be aligned to any database mentioned above were predicted by ESTScan [61] using Blast-predicted CDS as the model.

### 4.7. Differentially Expressed Genes

Expression data from two libraries (treatment and control) were determined by mapping to the transcriptome assembly using Bowtie2 software [62,63]. The fragments per kilobase of transcripts per million fragments mapped (FPKM) values were analysed further using RESM [64] to get differentially expressed genes (DEGs) in the spleen between the control and infected groups. Further, to determine the threshold *p*-value in multiple tests, a false discovery rate (FDR) was used. Furthermore, significant enrichment was calculated when FDR was <0.05 and FPKM values showed at least a two-fold difference between the two samples reads.

### 4.8. Real-Time Polymerase Chain Reaction

PCR primers were designed based on transcriptome sequences using Primer 2 Plus software (Table 2). cDNA was synthesised from 2 µg of total RNA using 200 U of M-MLV reverse transcriptase (Promega). β-Actin served as internal control and RT-qPCR was performed using iQSYBR Green Supermix (Bio-Rad Laboratories, Hercules, CA, USA), and each sample was run in triplicate. The thermal gradient feature (CFX96, Bio-Rad Laboratories) was used to determine the optimal annealing temperature for all primers. The real-time PCR program used was 95 °C for 3 min, followed by 40 cycles of 95 °C for 15 s, 58 °C for 15 s, and 72 °C for 35 s. Dissociation and melting curves of amplification products were performed and results were analysed using the CFX Manager Software package (Bio-Rad Laboratories). The 2^−ΔΔ*C*t^ method was chosen as the calculation method [65]. The difference in the cycle threshold (*C*_t_) value of the target gene and its housekeeping gene (*β-actin*), called Δ*C*_t_, was calculated using the following equation: ΔΔ*C*_t_ = (Δ*C*_t_ of bacterial challenge or PBS-injected group for the target gene at each time point) − (Δ*C*_t_ of the initial control).

### 4.9. Statistical Analyses

Statistical analyses were performed using SPSS 16.0 software. All data are given as mean ± SD. Significant differences between samples were analysed by one-way analysis of variance (ANOVA), and Duncan’s tests at a significance level of 0.05.

## 5. Conclusions

This study provides necessary information on differential immune gene transcriptome profiling in largemouth bass (*M. salmoides*) infected with *N*. *seriolae*. Moreover, this transcriptome assembly could be used as a reference for studies related to comparative biology within the genus or family. Of course, we acknowledge that this transcriptome-level response to *N. seriolae* infections is a preliminary study and larger scale studies are required to further understand the defence mechanisms in largemouth bass.

## Figures and Tables

**Table 1:** Immune-related differentially expressed genes (DEGs) regulated after infection.

Name	Description	Fold Change	Change
RIG I like receptor			
*trim25*	*Tripartite motif-containing protein 25*	1.32	Up
*dhx58*	*ATP-dependent RNA helicase dhx58*	3.41	Up
*ddx3x*	*ATP-dependent RNA helicase ddx3x*	6.28	Up
*ikbke*	*Inhibitor of nuclear factor κ-B kinase subunit epsilon*	2.54	Up
*ikbkg*	*Inhibitor of nuclear factor κ-B kinase subunit γ*	2.87	Up
*irf3*	*Interferon regulatory factor 3*	2.50	Up
*irf7*	*Interferon regulatory factor 7*	1.10	Up
*casp8*	*Caspase 8*	1.35	Up
*casp10*	*Caspase 10*	1.08	Up
*ikkβ*	*Inhibitor of nuclear factor κ-b kinase subunit β*	−1.62	Down
*traf6*	*TNF receptor-associated factor 6*	3.11	Up
*p38*	*p38 MAP kinase*	−8.64	Down
*il-8*	*Interleukin-8*	2.27	Up
*ip-10*	*Chemokine (c-x-c motif) 10*	2.32	Up
*tnf-α*	*Tumor necrosis factor superfamily, member 2*	2.32	Up
*il-12*	*Interleukin-12a*	3.41	Up
*lbp*	*Lipopolysaccharide-binding protein*	2.94	Up
*casp8*	*Caspase 8*	1.35	Up
*rip1*	*Receptor-interacting serine/threonine-protein kinase 1*	1.31	Up
*ctsk*	*Cathepsin K*	−11.52	Down
*tlr-3*	*Toll-like receptor 3*	1.16	Up
*ifnar1*	*Interferon receptor 1*	1.67	Up
*stat1*	*Signal transducer and activator of transcription 1*	2.38	Up
*il1b*	*Interleukin 1, β*	2.44	Up
*rantes*	*Chemokine (c-c motif) 5*	2.03	Up
*cd40*	*Tumor necrosis factor receptor superfamily, member 5*	1.15	Up
*cd86*	*cd86 antigen*	−1.22	Down
Cytokine-cytokine receptor interaction			
*cxcl7*	*Platelet basic protein*	−1.51	Down
*cxcl10*	*Chemokine (c-x-c motif) 10*	2.27	Up
*cxcl13*	*Chemokine (c-x-c motif) 13*	2.27	Up
*cxcl14*	*Chemokine (c-x-c motif) 14*	−2.59	Down
*il8rb*	*Interleukin 8 receptor, β*	−1.16	Down
*il8ra*	*interleukin 8 receptor, α*	−1.16	Down
*cxcr3*	*Chemokine (c-x-c receptor) type 3*	1.13	Up
*xcr1*	*Chemokine xc receptor 1*	3.82	Up
*ccl20*	*Chemokine (c-c motif) 20*	5.16	Up
*ccl25*	*Chemokine (c-c motif) 25*	−1.17	Down
*ccl19*	*Chemokine (c-c motif) 19*	5.16	Up
*ccl21*	*Chemokine (c-c motif) 21*	−1.17	Down
*ccl5*	*Chemokine (c-c motif) 5*	2.03	Up
*ccr3*	*Chemokine (c-c receptor) type 3*	3.82	Up
*il6r*	*Interleukin 6 receptor*	−2.11	Down
*il11ra*	*Interleukin 11 receptor α*	4.20	Up
*csfr*	*Colony-stimulating factor receptor (granulocyte)*	1.76	Up
*il13ra1*	*Interleukin 13 receptor, α-1*	2.91	Up
*il12rb2*	*Interleukin 12 receptor, β-2*	1.76	Up
*il23r*	*Interleukin 23, receptor*	1.76	Up
*csf2ra*	*Granulocyte-macrophage colony-stimulating factor receptor α*	2.91	Up
*il1ra*	*Interleukin 1 receptor, α*	1.15	Up
*il21r*	*Interleukin 21, receptor*	−2.67	Down
*epor*	*Erythropoietin receptor*	−1.85	Down
*ghr*	*Growth hormone receptor*	−9.49	Down
*mpl*	*Thrombopoietin receptor*	−1.26	Down
*flt1*	*FMS-like tyrosine kinase 1*	1.18	Up
*met*	*Proto-oncogene tyrosine-protein kinase met*	−2.17	Down
*egf*	*Epidermal growth factor*	−1.07	Down
*egfr*	*Epidermal growth factor receptor*	−1.64	Down
*csf1r*	*Macrophage colony-stimulating factor 1 receptor*	1.80	Up
*ifnar1*	*Interferon receptor, 1*	1.67	Up
*ifnar2*	*Interferon receptor, 2*	1.52	Up
*il10ra*	*Interleukin 10 receptor, α*	4.20	Up
*il10rb*	*Interleukin 10 receptor, β*	−1.50	Down
*tnfsf11b*	*Tumor necrosis factor receptor superfamily, member 11B*	1.80	Up
*tnfsf12*	*Tumor necrosis factor ligand superfamily, member 12*	−1.10	Down
*Tnfb*	*Tumor necrosis factor b (TNF superfamily, member 2)*	2.33	Up
*tnfsf14*	*Tumor necrosis factor (receptor) superfamily, member 14*	1.20	Up
*tnfsf6b*	*Tumor necrosis factor (receptor) superfamily, member 6b*	1.80	Up
*faslg*	*Tumor necrosis factor (ligand) superfamily, member 6*	1.13	Up
*cd40*	*Tumor necrosis factor (receptor) superfamily, member 5*	1.15	Up
*tnfsf13b*	*Tumor necrosis factor (ligand) superfamily, member 13B*	−1.19	Down
*tgfbr2*	*TGF-β receptor type-2*	−2.10	Down
Antigen processing and presentation			
*psme1*	*Proteasome activator subunit 1*	1.57	Up
*hsp70*	*Heat shock 70 kDa protein*	4.01	Up
*hsp90*	*Molecular chaperone HtpG*	2.00	Up
*tap1/2*	*ATP-binding cassette, subfamily b (MDR/TAP), member 2*	2.72	Up
*tapbp*	*Tap binding protein (tapasin)*	2.46	Up
*pdia3*	*Protein disulfide isomerase family a, member 3*	10.67	Up
*mhci*	*Major histocompatibility complex, class I*	5.19	Up
*b2m*	*β-2-microglobulin*	1.25	Up
*mhcii*	*Major histocompatibility complex, class II*	1.99	Up
*ciita*	*Class II, major histocompatibility complex, transactivator*	1.74	Up
*tcr-α*	*T cell receptor α chain v region*	−9.97	Down
Natural Killer Cell Mediated Cytotoxity			
*cd48*	*cd48 antigen*	2.29	Up
*trailr*	*Tumor necrosis factor (receptor) superfamily, member 10*	−1.02	Down
*prf1*	*Perforin 1*	9.91	Up
*grb*	*Granzyme B*	−1.74	Down
*igg*	*Immunoglobulin heavy chain g*	5.30	Up
*fcγr3*	*Low affinity immunoglobulin γ Fc region receptor III*	1.59	Up
*fasl*	*Tumor necrosis factor (ligand) superfamily, member 6*	1.13	Up
*shp-2*	*Tyrosine-protein phosphatase non-receptor type 11*	9.78	Up
*dap-12*	*Tyro protein tyrosine kinase binding protein*	−2.26	Down
*vav1*	*Guanine nucleotide exchange factor vav*	1.24	Up
*3bp2*	*sh3-domain binding protein 2*	9.78	Up
*slp-76*	*Lymphocyte cytosolic protein 2*	−3.12	Down
*shc1*	*SHC-transforming protein 1*	9.78	Up
*can*	*Serine/threonine-protein phosphatase 2B catalytic subunit*	1.58	Up
*pkc*	*Classical protein kinase c α type*	1.0	Up

**Table 2:** Primer name, sequence, target gene, and their application used in the present study.

Name	Sequence	Target Gene	Application
LMBIL-12 F1Q	TCTTCCATCCTTGTGGTCTTCC	*IL-12p40*	qPCR
LMBIL-12 R1Q	CAGTTCCAGGTCAAAGTGGTC		
LMBIL-8 F1Q	GAGCCATTTTTCCTGGTGACT	*IL-8*	
LMBIL-8 R1Q	TCCTCATTGGTGCTGAAAGATC		
LMBIL-1 F1Q	CAAGATGCCTAAGGGACTGGA	*IL-1*	
LMBIL-1 R1Q	AGGTGAACTTTGCGGTTCTC		
LMBTCR F1Q	ATCATCTTTGGAAGTGGAACC	*TCR*	
LMBTCR R1Q	GATGTTGAAGACGACGGTCTT		
LMBCD40 F1Q	TACAAGTGAAACATGGGGCAAC	*CD40*	
LMBCD40 R1Q	TGATGAAGAGTCCACCTTACCG		
LMBβ-Actin375F	CCACCACAGCCGAGAGGGAA	*β-actin*	
LMBβ-Actin375R	TCATGGTGGATGGGGCCAGG		
LMBIL-1βF	TTGCCATAGAGAGGTTTA	*IL-1β*	
LMBIL-1βR	ACACTATATGCTCTTCCA		
LMBTNFα-F	CTAGTGAAGAACCAGATTGT	*TNF-α*	
LMBTNFα-R	AGGAGACTCTGAACGATG		

## Supplementary Materials

Supplementary materials can be found at www.mdpi.com/1422-0067/17/8/1315/s1.

## Abbreviations

DGE: digital gene expression
Tgicl: is a pipeline for analysis of large Expressed Sequence Tags (EST)
NR: NCBI non-redundant protein
NT: NCBI nucleotide
COG: Clusters of Orthologous Groups
KEGG: Kyoto Encyclopedia of Genes and Genomes
ORF: open reading frame
KO: KEGG Orthology
CDS: coding region of protein
INDEL: insertion and deletion
*HISAT*: is a fast and sensitive spliced alignment program for mapping RNA-Seq reads
GATK: The Genome Analysis Toolkit
FPKM: fragments per kilobase of transcripts per million fragments mapped
RESM: is a software package for estimating gene and isoform expression levels from RNA-Seq data
FDR: False discovery rate
NLR: NOD-like receptors
TLR: Toll-like receptors
PAMP: Pathogen-associated molecular patterns
PRR: Pattern-recognition receptors
CLR: C-type lectin receptors
SRA: NCBI Sequence Read Archive
SSR: Simple Sequence Repeat
SNP: single nucleotide polymorphism
