# Supplementary material for: De Novo Transcriptome Analysis of Differential Functional Gene Expression in Largemouth Bass (Micropterus salmoides) after Challenge with Nocardia seriolae
Source: Int J Mol Sci. 2016 Aug 11;17(8):1315. doi: 10.3390/ijms17081315 (PMC5000712; doi:10.3390/ijms17081315)
Supplement: Supplementary file 1 [file ijms-17-01315-s001.pdf]

Supplementary Materials: De Novo Transcriptome Analysis of Differential Functional Gene Expression in Largemouth Bass (*Micropterus salmoides*) after Challenge with *Nocardia seriolae*

Omkar Byadgi, Chi-Wen Chen, Pei-Chyi Wang, Ming-An Tsai and Shih-Chu Chen

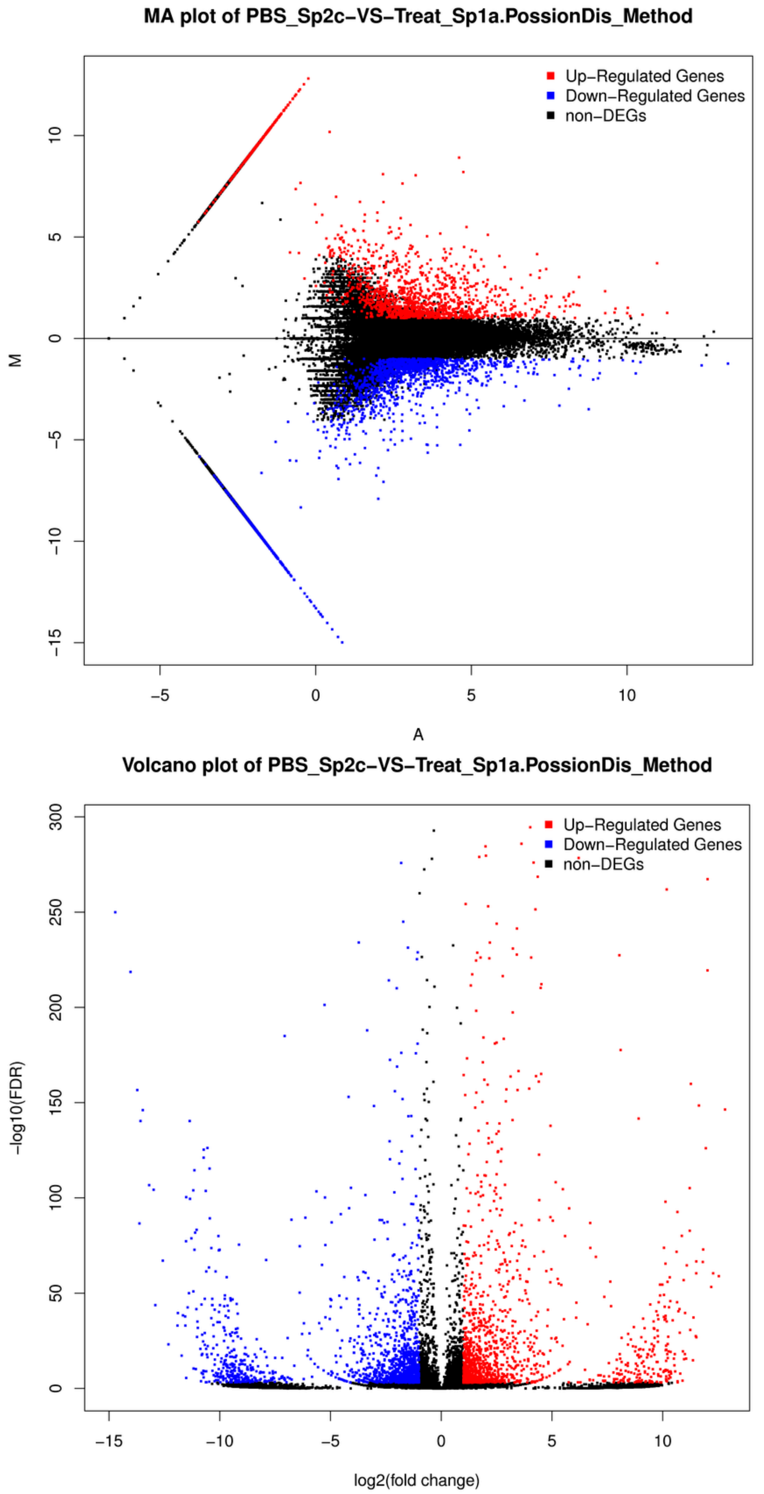

Figure S1. Volcano plot of differentially expressed genes identified between the control and bacterial challenged groups.

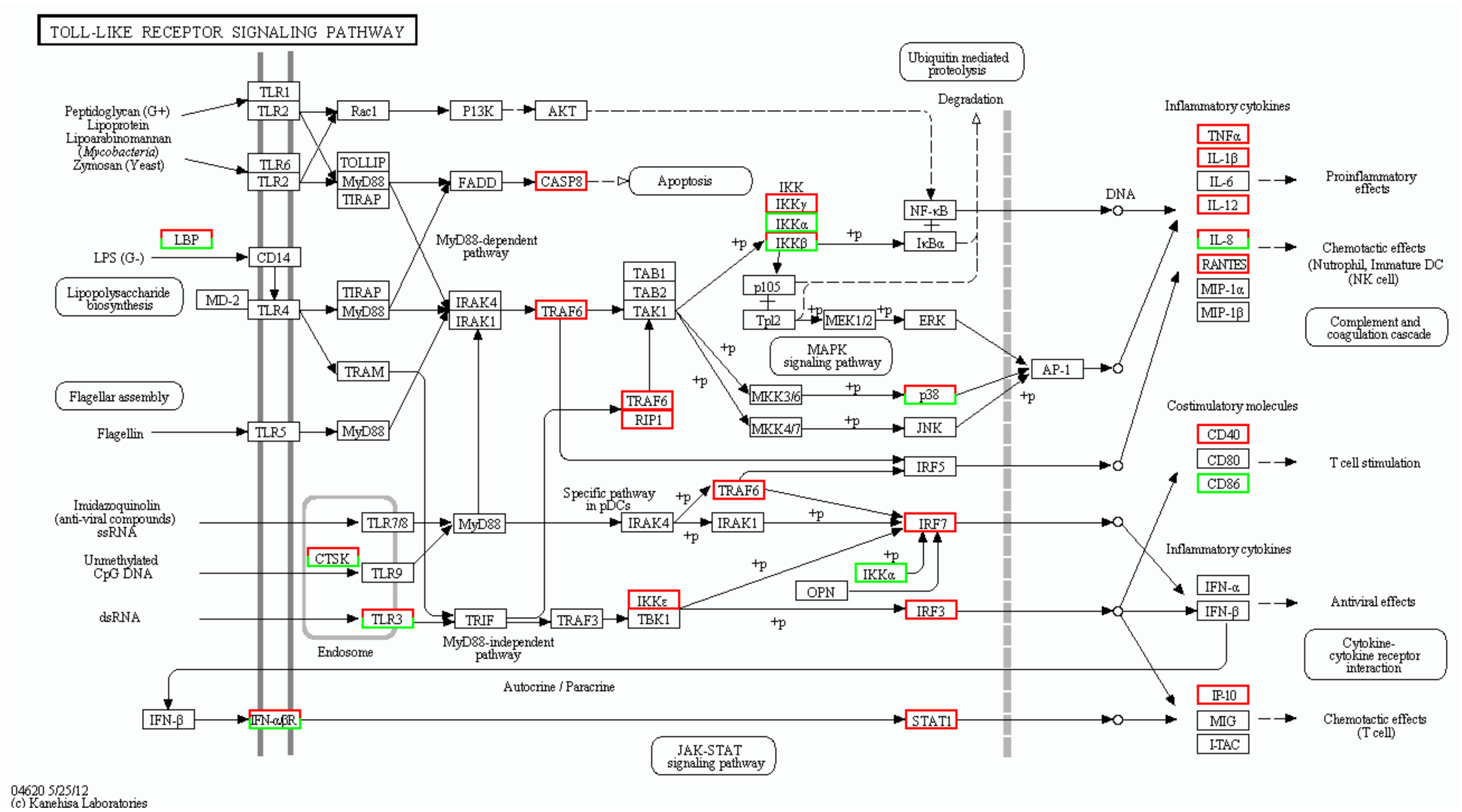

Figure S2. Toll-like receptor signaling pathway mapping by KEGG. Red boxes indicate significantly differentially upregulated expression and green boxes indicate unchanged expression in the transcriptomic profile.

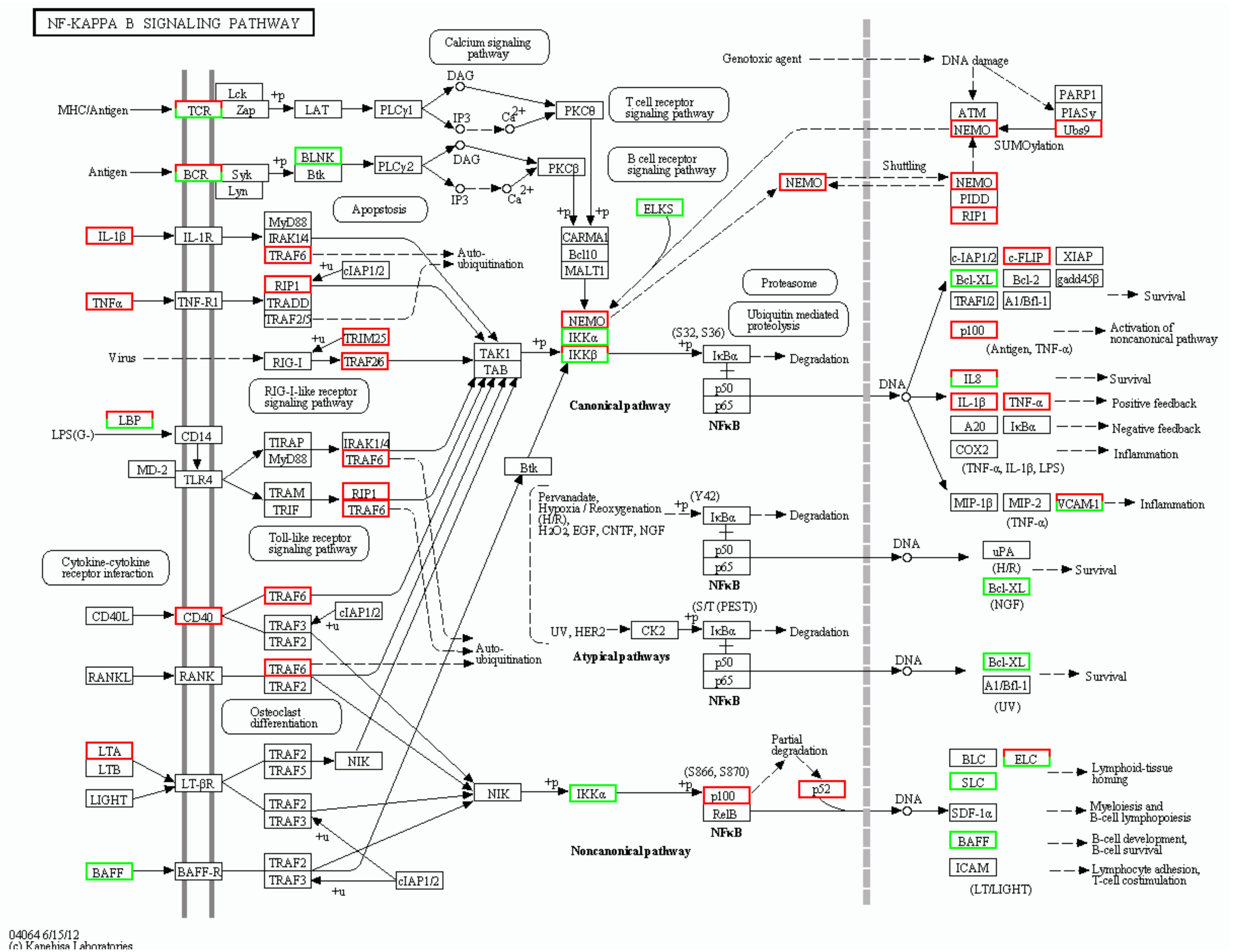

Figure S3. NF-κB signaling pathway mapping by KEGG. Red boxes indicate significantly differentially upregulated expression and green boxes indicate unchanged expression in the transcriptomic profile.

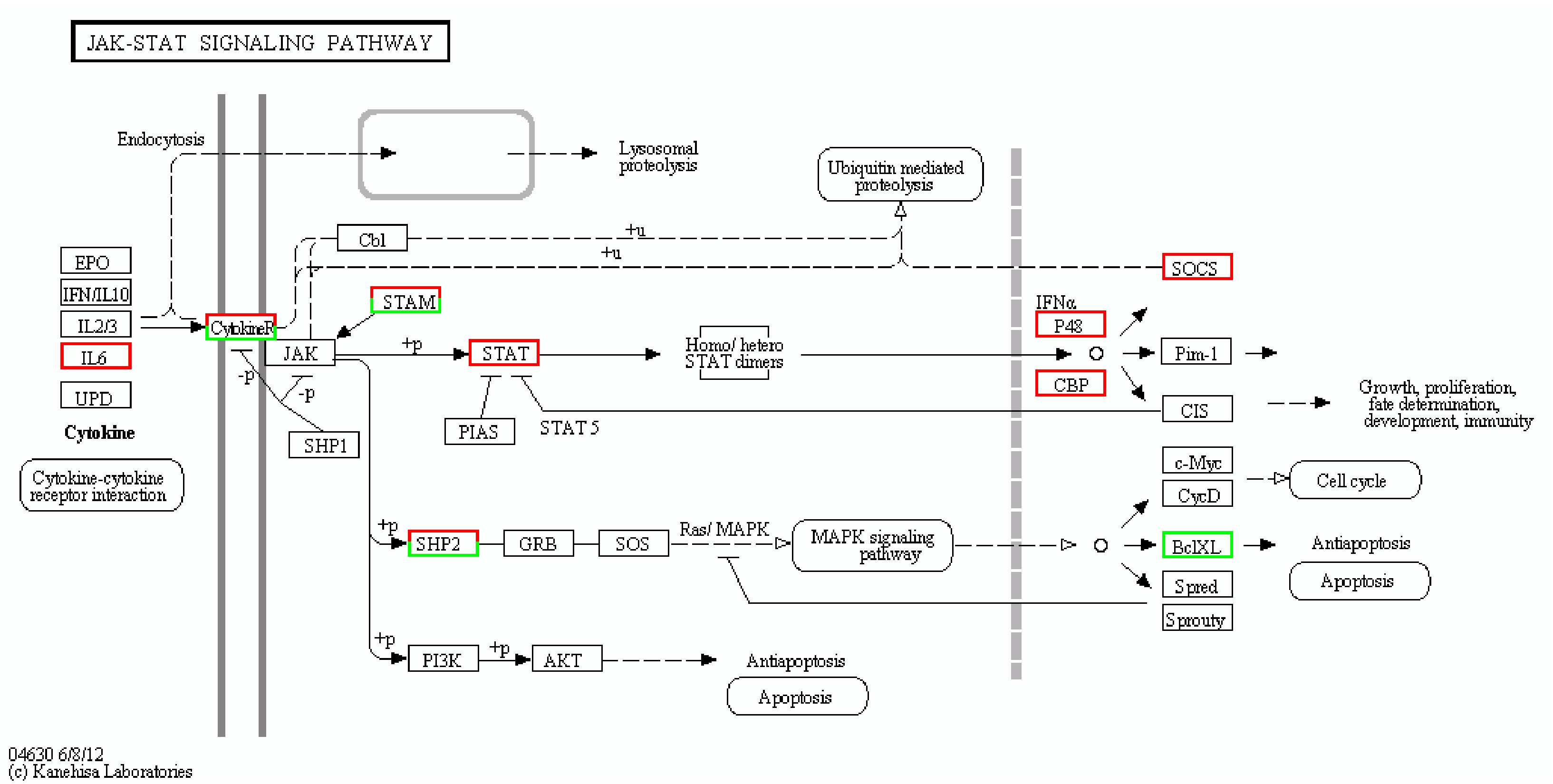

Figure S4. JAK STAT signaling pathway mapping by KEGG. Red boxes indicate significantly differentially upregulated expression and green boxes indicate unchanged expression in the transcriptomic profile.

Table S1. Length distribution of assembled transcripts and unigenes.

	Sample	Total Number	Total Length	Mean Length	N50	GC %
Assembled transcript	PBS	61,327	47,805,064	779	1472	45.85
	Treatment	57,949	45,818,753	790	1496	45.96
Unigenes	PBS	48,236	41,881,916	868	1571	45.96
	Treatment	45,293	39,750,518	877	1607	46.13
	Merged unigenes	47,881	49,734,288	1038	1983	45.94

Table S2. Summary of annotations of assembled unigenes.

Anno_Database	Annotated Number
Nr	28,964
Nt	36,686
Swissprot	24,830
KEGG	20,329
COG	8913
Interpro	22,194
GO	835
All annotated	37,712
All sequence	47,881

Table S3. Analysis of immune relevant pathway.

Pathway	Number of DEG			All Genes with Pathway Annotation	*q*-Value	Pathway ID
	Up	Down	Total DEGs			
Natural killer cell mediated cytotoxicity	30	15	45 (2.33%)	292 (1.44%)	7.22E-03	ko04650
Hematopoietic cell lineage	29	22	51 (2.64%)	236 (1.16%)	2.20E-06	ko04640
Toll-like receptor signaling pathway	29	10	39 (2.02%)	214 (1.05%)	1.36E-03	ko04620
Fc γ R-mediated phagocytosis	24	10	34 (1.76%)	433 (2.13%)	1.00E+00	ko04666
Antigen processing and presentation	23	13	33 (1.71%)	179 (0.88%)	2.18E-03	ko04612
NOD-like receptor signaling pathway	22	17	39 (2.02%)	265 (1.3%)	2.68E-02	ko04621
Chemokine signaling pathway	22	34	56 (2.9%)	486 (2.39%)	2.90E-01	ko04062
Leukocyte transendothelial migration	21	25	46 (2.38%)	412 (2.03%)	4.53E-01	ko04670
RIG-I-like receptor signaling pathway	20	15	35 (1.81%)	144 (0.71%)	9.40E-06	ko04622
Complement and coagulation cascades	15	17	32 (1.65%)	141 (0.69%)	9.00E-05	ko04610
Cytosolic DNA-sensing pathway	13	9	22 (1.14%)	113 (0.56%)	7.22E-03	ko04623
Intestinal immune network for IgA production	11	16	27 (1.4%)	144 (0.71%)	4.50E-03	ko04672
T cell receptor signaling pathway	10	14	24 (1.24%)	339 (1.67%)	1.00E+00	ko04660

Table S4. Important immune related genes identified from DEG in largemouth bass.

Unigenes	Control	Treatment	Log2 Fold Change	NCBI GenBank No.	Species	Identity	Description
Unigene18576_All	4.48	17.84	1.99	XM_005471524.1	Tilapia	74	Major histocompatibility complex, class II
Unigene38513_All	0.84	30.68	5.19	HM236159.1	Mi-iuy croaker	75	Major histocompatibility complex, class I
CL167.Contig8_All	0.01	1.04	6.70	XM_008279960.1	Bicolor Damselfish	99	ATP-dependent RNA helicase DDX3X
Unigene36287_All	0.68	7.23	3.41	KJ818334.1	European seabass	83	Interleukin 12A
Unigene20650_All	16.47	42.07	1.35	XM_010754586.1	European seabass	79	Caspase 8
Unigene2531_All	8.08	85.98	3.41	KR062119.1	Japanese seabass	84	ATP-dependent RNA helicase DHX58
CL1338.Contig2_All	2.07	10.03	2.28	KJ818344.1	European seabass	80	Interleukin 8 (CXC chemokine)
Unigene311_All	4.32	24.54	2.51	KP409190.1	Mandarin fish	91	Interferon regulatory factor 3
Unigene9993_All	6.31	13.61	1.11	AY647434.1	Mandarin fish	89	Interferon regulatory factor 7
Unigene6357_All	11.66	47.83	2.04	GU181392.1	Gilthead seabream	73	C-C motif chemokine 5
CL2369.Contig1_All	28.13	147.42	2.39	FJ629185.1	Mandarin fish	95	Signal transducer and activator of transcription 1
CL669.Contig4_All	7.78	126.2	4.02	JX500752.1	Mandarin fish	94	Heat shock 70 kDa protein
Unigene535_All	24.97	54.08	1.11	EF596785.1	Mandarin fish	75	T cell receptor alpha chain V region
CL1338.Contig2_All	2.07	10.03	2.28	XM_004555687.3	zebra mbuna	69	C-X-C motif chemokine 10
Unigene14937_All	4.18	14.05	1.75	KR921863.1	Mi-iuy croaker	82	Nucleotide-binding oligomerization domain-containing protein 1
CL1024.Contig2_All	0.01	17.3	10.76	JX976626.1	Gilthead seabream	68	Interleukin 18
CL73.Contig4_All	10.11	26.34	1.38	XM_010729260.1	Large yellow croaker	77	Interleukin 8 receptor beta
Unigene1492_All	7.92	156.7	4.31	XM_010729149.1	Large yellow croaker	82	Suppressor of cytokine signaling 1
Unigene32804_All	0.46	18.15	5.30	AY885707.1	Orange spotted grouper	82	Immunoglobulin heavy chain
CL482.Contig4_All	2.73	7.17	1.39	XM_010732863.1	Large yellow croaker	88	Mothers against decapentaplegic homolog 1
CL1967.Contig2_All	7.05	15.45	1.13	XM_010739771.1	Large yellow croaker	80	Tumor necrosis factor ligand superfamily member 6
Unigene12232_All	11.33	22.71	1.00	EF492047.1	Mandarin fish	92	Nuclear factor NF-κ-B p105 subunit
